# Morphometry of eyes, antennae and wings in three species of *Siagona*(Coleoptera, Carabidae)

**DOI:** 10.3897/zookeys.100.1528

**Published:** 2011-05-20

**Authors:** Federica Talarico, Pietro Brandmayr, Anita Giglio, Alessandro Massolo, Tullia Zetto Brandmayr

**Affiliations:** 1 Department of Ecology, University of Calabria, I-87036; 2 Department of Ecosystem & Public Health, Calgary, Alberta, Canada

**Keywords:** Compound eyes, morphometric measurements, *Siagona*

## Abstract

In carabid beetles, physiological and behavioural characteristics reflect specific habitat demands and there is a strong correlation between body form and habit in species with different life style. In this study, we compared the morphometry and compound eye characteristics of three species of the genus *Siagona*: *Siagona jenissoni*, *Siagona dejeani* and *Siagona europaea*. These carabids have a stenotopic lifestyle in Mediterranean clayey soils, inhabiting the ground fissure system formed during the dry season. All species have a Mediterranean distribution and are nocturnal olfactory hunters, and are strict ant predators. For morphometric measurements, we considered body length (mm), wing length (mm), antenna length (mm), head width (mm), trochanter length (mm), number of ommatidia, eye surface area (mm2), ommatidia density (number of ommatidia/mm2 of eye surface area), head height (mm), thorax height (mm) and abdomen height (mm). The data revealed intersexual and interspecific differences. The three species differ in relative length of the antennae, density and number of ommatidia and relative trochanter length. Significant differences occurred in wing sizes, which are well developed in *Siagona europaea*, the only species capable of flight. When eye size is compared with other ground beetles of various lifestyles, *Siagona* shows pronounced “microphthalmy” an adaptation to subterranean life in clayey crevices of tropical and subtropical climates with a marked dry season.

## Introduction

Carabid beetles vary in body form and size, annual rhythmicity and habitat choice. They also differ in many physiological and behavioural characteristics that reflect specific habitat demands (Thiele 1977; Den Boer 1986). As a consequence there is a strong correlation between body form and habit (e.g., feeding, locomotion, burrowing and flying) in carabid beetles with different lifestyles. Those living in restricted or confined habitats, such as fissures in the ground or burrows, tend to have narrower and flatter (shallower) bodies, with the prothorax similar in width to the hind body. It has been suggested that this type of body form minimizes friction by causing less obstruction when moving through confined spaces (Forsythe 1982, 1983, 1991; Evans and Forsythe 1985).

Moreover, most ground beetles with seemingly similar body shapes have species-specific morphological peculiarities that reflect the special demands of their niches (Bauer and Kredler 1993). For example, although variability in eye morphology may be small among closely related species, e.g. those of the same genus, variability do exist if there are divergent habitat preferences (Bauer et al. 1998). The morphometry and eye morphology in three species of the genus *Carabus* (*Carabus coriaceus*, *Carabus lefebvrei* and *Carabus presli*) were recently investigated in relation to habitat demands (Talarico et al. 2007). The three species are large-spectrum olfactory hunters, but their different lifestyles have influenced body and eye characteristics: the number of ommatidia is significantly higher in *Carabus coriaceus* than in *Carabus lefebvrei* and *Carabus preslii*, and the authors suggested that this could be habitat-related. As a consequence, *Carabus coriaceus* and *Carabus lefebvrei* can be included in the second group of Bauer and Kredler (1993), including species with no preferred activity period (i.e., active by day and night, but preferably at twilight), while *Carabus preslii* belongs to the third group of nocturnal species.

The genus *Siagona* (tribe Siagonini) should be placed among the less derived Carabidae (Carabinae, Caraboidea Simplicia of Jeannel 1941, the so called “lower carabids”), and a recent study by Ball and Shpeley (2005) reported marked differences from the very similar genus *Cymbionotum* Baudi di Selve.

The biology of siagonines is poorly known. Andrewes (1929) hypothesized relationships between the Siagonini tribe and termites. The same author collected adult beetles of four *Siagona* species from India during the rainy season in vegetable refuse surrounding rice fields. The genus has a wide geographic distribution, including India, Arabia, Africa and the Mediterranean region. Three species are present in Southern Europe, *Siagona jenissoni* Dejean, 1826, *Siagona dejeani* Rambur, 1837 and *Siagona europaea* Dejean, 1826 (Bauer et al. 2005). All siagonines have a strikingly flat body with a stalk-like constriction between the pro- and mesothorax and strong mandibles with large retinaculum. The flatness of the body and thoracic constriction are possible adaptations to life in narrow soil crevices. The short but strong mandibles are well-suited for grasping and chewing arthropod prey with tough and flexible cuticles (e.g. ants; Zetto Brandmayr and Pizzolotto 1994; Bauer et al. 2005).

*Siagona europaea* is exclusively myrmecophagous, both of adult ants and their brood (Zetto Brandmayr and Pizzolotto 1994; Zetto Brandmayr et al. 1998; Bauer et al. 2005). It has a Mediterranean distribution (Italy, Spain, Greece and North Morocco), preferring open, sclerophyllous habitats of the Mediterranean biome (Brandmayr and Pizzolotto 1990). In Southern Italy, *Siagona europaea* occurs in pastures and abandoned fields only in clayey soils up to an altitude of about 250 m a.s.l., while in Calabria it occurs up to ca. 450 m (Pizzolotto et al. 2005). In early spring, when soil moisture is high, the beetles are found under stones. From mid-April onwards, when the soil dries out and becomes deeply fissured, they retreat into deeper crevices, especially during the hot and dry hours of the day. Their activity is mainly nocturnal, as shown by recordings and by the structure of their compound eyes (Bauer et al. 2005), with a value of ommatidia/mm body length typical for nocturnal species (cf. Bauer and Kredler 1993).

Only fragmentary information is available for *Siagona jenissoni* and *Siagona dejeani*. They occur in southern Spain, between Cadiz and Malaga, in Portugal (Serrano 2003) and on the coast of Morocco (Andrewes 1929; Antoine 1955). The aim of this study was to acquire further knowledge on the biology of the three southern European *Siagona* species by morphometric investigations of intersexual and interspecific differences of some morphological features, such as the antennae, eyes and wings.

## Methods

### Animals

The sample consisted of 20 individuals (10 males and 10 females) for each species: *Siagona jenissoni*, *Siagona dejeani* and *Siagona europaea*. Specimens of *Siagona europaea* were collected in southern Italy (Calabria, Squillace, Catanzaro, 250 m a.s.l.) mostly by bait-traps in open fields and pastures during the spring of 2004, while *Siagona jenissoni* and *Siagona dejeani* were collected in southern Spain (Andalusia, between Algesiras and Cadiz) in March of 2005 (100–400 m a.s.l.).

### Morphometric analyses

The animals were stored in alcohol (70%). Photographs were taken with a stereoscope (Zeiss Stemi SV 11Apo) and acquired by Matrox PC-VCR software (for Windows® 2000). For each individual, we measured body length (mm), wing length (mm), antenna length (mm), head width (mm), trochanter length (mm), number of ommatidia, eye surface area (mm2), ommatidia density (number of ommatidia/mm2 of eye surface area), head height (mm), thorax height (mm) and abdomen height (mm).

Relative measures of antennal lengths, number of ommatidia and eye surface area were weighted against head width, while trochanter length, head height, thorax height and abdomen height were weighted against body length. To determine the number of ommatidia and cornea size, we softened the specimens in hot potash lye for a few minutes. The cornea was removed and fixed through the following stations: distilled water, acetone, ethanol (70%), absolute ethanol and xylol. It was then spread on a microscope slide and photographed. Measurements were taken using Sigma Scan Pro 5 Software (SPSS® Inc.).

### Statistical analyses

Sexual dimorphism in each species was tested using the Mann-Whitney U test (Siegel and Castellan 1988), while the Kruskall-Wallis test was used to test for morphological differences among species (Sokal and Rohlf 1995). Pairwise comparisons (between species) were performed with the Mann-Whitney U test, and significance levels were corrected using the Dunn-Šidák significance level correction method: *α’*= 1 – (1 – *α*)1/*k*, were *k* is the number of comparisons (Sokal and Rohlf 1995).

The probability level was computed using a complete randomisation method (permutation or exact test; *Pexact*) or by a Monte Carlo simulation based on 10 000 sampled tables (*PMonteCarlo*) when computation was not possible (Mehta and Patel 1996; Good 2000).

The multivariate general lineal model (GLM) with species and sex as main factors was applied to sensorial structures, eye asymmetry and main body size measures to verify previously performed univariate hypothesis testing. Multivariate differences between factors were tested by Pillai’s Trace, while univariate tests were computed using the type III sum of squares.

Means are reported with standard error of means (± SEM) throughout the text.

Statistical analyses were performed using the Statistical Package for Social Sciences 13.01 (SPSS® Inc.).

## Results

**Figure 1.**
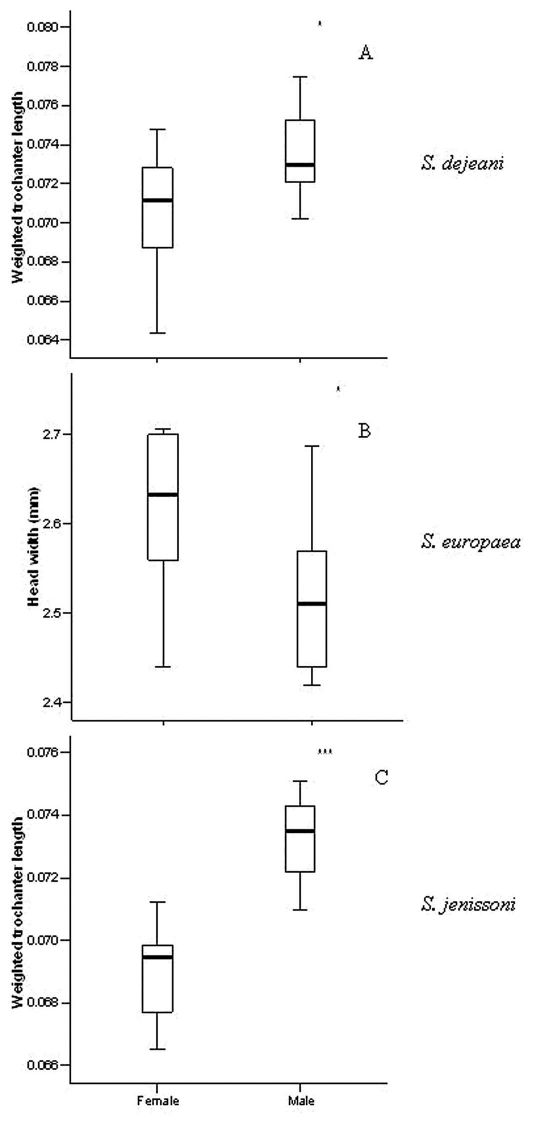
Measured traits between sexes in the three species: **A** weighted trochanter length (mm) in *Siagona dejeani* **B** head width (mm) in *Siagona europaea* **C** weighted trochanter length (mm) in *Siagona jenissoni*.

The three species presented some sex differences related to size (Table 1, Fig. 1). Males of *Siagona dejeani* and *Siagona jenissoni* had significantly longer trochanters (relative to body length) compared to females (respectively *U* = 21.5, *W* = 76.5, *PExact* = 0.029 and *U* = 1.0, *W* = 56.0, *PExact* < 0.001), while in *Siagona europaea* females had wider heads than males (*U* = 23.0, *W* = 78.0, *PExact* = 0.043). Notably, there was no difference in the size of sensory structures (antennae and eyes) (*PExact* > 0.05); therefore, we evaluated specific differences in sensory structures with no concern for gender.

**Table 1.** Sex differences in body and eye morphological characteristics (means and Standard Error of Means) in three species of *Siagona*. Mann-Whitney test results are shown, with significance levels estimated using a permutation procedure (*PExact*). Statistically significant results are in bold. L = left, R = right.<br/>

*Species*		*Gender*				*Mann-Whitney test*		
		*Female*		*Male*				
		*Mean*	*SEM*	*Mean*	*SEM*	*U*	*W*	**P**Exact
*Siagona dejeani*	Body length (mm)	23.33	0.16	22.83	0.29	30.0	85	0.143
	Antenna length (mm)	12.58	0.15	12.82	0.25	43.0	98	0.631
	Head width (mm)	5.06	0.06	5.09	0.09	46.5	101.5	0.796
	Number of ommatidia L	407.80	25.21	404.10	14.77	49.0	104	0.971
	Number of ommatidia R	404.30	12.47	356.40	15.87	22.0	77	*0.035*
	Eye surface L (mm2)	0.06	0.01	0.06	0.01	41.0	96	0.529
	Eye surface R (mm2)	0.08	0.00	0.07	0.00	33.0	88	0.218
	Trochanter length (mm)	1.65	0.02	1.71	0.04	39.0	94	0.436
	Head height (mm)	2.80	0.08	2.76	0.08	44.5	99.5	0.684
	Thorax height (mm)	3.37	0.06	3.33	0.06	44.5	99.5	0.684
	Abdomen height (mm)	3.22	0.13	3.17	0.11	44.5	99.5	0.684
	Weighted antenna length	2.49	0.04	2.52	0.05	42.0	97	0.579
	Weighted trochanter length	0.07	0.00	0.07	0.00	21.5	76.5	*0.029*
	Weighted ommatidia L number	80.86	5.30	79.51	2.89	49.0	104	0.971
	Weighted ommatidia R number	80.03	2.68	69.95	2.65	15.0	70	*0.007*
	Weighted head height	0.12	0.00	0.12	0.00	49.0	104	0.971
	Weighted thorax height	0.14	0.00	0.15	0.00	48.0	103	0.912
	Weighted abdomen height	0.14	0.01	0.14	0.00	49.0	104	0.971
	Right ommatidia density	5019.71	252.49	4857.98	162.19	48.0	103	0.912
	Left ommatidia density	19964.03	9939.31	20421.65	10096.35	43.0	98	0.631
*Siagona europaea*	Body length (mm)	11.73	0.22	11.16	0.18	26.5	81.5	0.075
	Antenna length (mm)	6.96	0.16	7.03	0.11	50	105	1.000
	Head width (mm)	2.64	0.04	2.52	0.03	23	78	*0.043*
	Number of ommatidia L	528.60	28.98	564.90	25.03	40	95	0.481
	Number of ommatidia R	494.10	31.81	536.10	17.72	36	91	0.315
	Eye surface L (mm2)	0.01	0.00	0.01	0.00	46	101	0.796
	Eye surface R (mm2)	0.01	0.00	0.01	0.00	48.5	103.5	0.912
	Trochanter length (mm)	0.85	0.02	0.84	0.02	39	94	0.436
	Head height (mm)	1.36	0.05	1.25	0.04	27	82	0.089
	Thorax height (mm)	1.71	0.05	1.71	0.03	46.5	101.5	0.796
	Abdomen height (mm)	1.84	0.05	1.76	0.07	35.5	90.5	0.280
	Weighted antenna length	2.65	0.06	2.79	0.04	31	86	0.165
	Weighted trochanter length	0.07	0.00	0.08	0.00	37	92	0.353
	Weighted ommatidia L number	200.85	10.92	223.42	8.45	32	87	0.190
	Weighted ommatidia R number	187.94	12.24	212.58	7.25	32	87	0.190
	Weighted head height	0.12	0.01	0.11	0.00	47	102	0.853
	Weighted thorax height	0.15	0.01	0.15	0.00	28	83	0.105
	Weighted abdomen height	0.16	0.01	0.16	0.01	49	104	0.971
	Right ommatidia density	64068.08	9474.82	71412.86	7665.16	44	99	0.684
	Left ommatidia density	68827.82	8122.49	68715.15	5377.99	40	95	0.481
*Siagona jenissoni*	Body length (mm)	14.38	0.14	14.11	0.09	35	90	0.280
	Antenna length (mm)	8.89	0.08	8.97	0.09	41.5	96.5	0.529
	Head width (mm)	3.20	0.04	3.31	0.03	25	80	0.063
	Number of ommatidia L	314.90	11.88	316.30	3.75	48	103	0.912
	Number of ommatidia R	346.50	9.23	363.00	15.99	42.5	97.5	0.579
	Eye surface L (mm2)	0.04	0.00	0.04	0.00	45.5	100.5	0.739
	Eye surface R (mm2)	0.04	0.00	0.03	0.00	41.5	96.5	0.529
	Trochanter length (mm)	0.99	0.01	1.03	0.01	23.5	78.5	*0.043*
	Head height (mm)	1.82	0.06	1.74	0.06	44	99	0.684
	Thorax height (mm)	2.09	0.06	2.20	0.07	38.5	93.5	0.393
	Abdomen height (mm)	2.24	0.13	2.28	0.12	48	103	0.912
	Weighted antenna length	2.79	0.03	2.71	0.03	24	79	*0.052*
	Weighted trochanter length	0.07	0.00	0.07	0.00	1	56	*<0.001*
	Weighted ommatidia L number	98.82	4.35	95.57	1.12	36	91	0.315
	Weighted ommatidia R number	108.83	3.87	109.71	4.89	49	104	0.971
	Weighted head height	0.13	0.01	0.12	0.00	50	105	1.000
	Weighted thorax height	0.15	0.01	0.16	0.00	31	86	0.165
	Weighted abdomen height	0.16	0.01	0.16	0.01	45	100	0.739
	Right ommatidia density	10786.08	1392.59	11925.89	1320.63	42	97	0.579
	Left ommatidia density	8943.47	1037.65	9130.10	821.02	43	98	0.631

**Figure 2.**
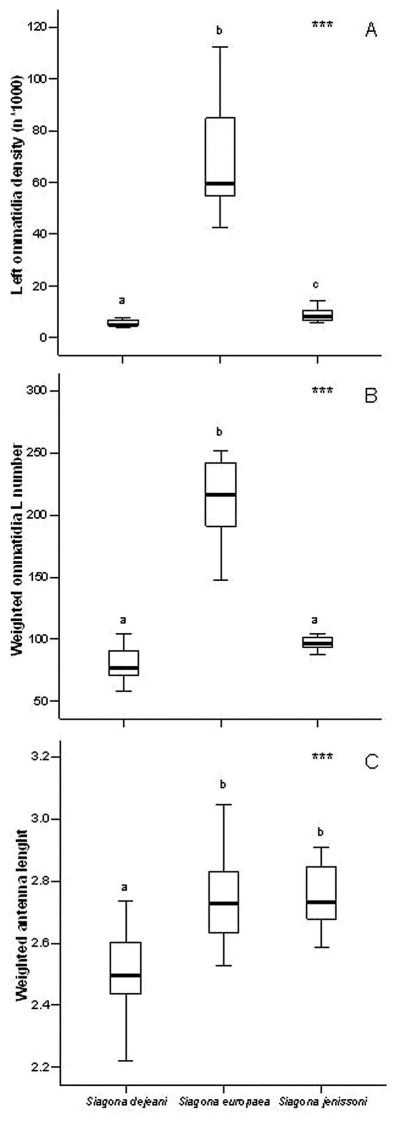
Measured traits: **A** ommatidia density (N/mm2) **B** weighted ommatidia number **C** weighted antenna length.

Ommatidia density differed significantly among species (*X2* = 30.951, d.f. = 2, *PExact* < 0.001), being significantly higher (*PExact* < 0.05) in *Siagona europaea* and lower in *Siagona dejeani* (Table 2, Fig. 2A). The weighted number of ommatidia in *Siagona europaea* was higher (*X2* = 45.057, d.f. = 2, *PExact* < 0.001), but there was no significant difference between *Siagona dejeani* and *Siagona jenissoni* (Fig. 2B). *Siagona dejeani*’s antennae were significantly shorter than those of the other two species (*X2* = 24.521, d.f. = 2, *PExact* < 0.001) (Fig. 2C).

The GLM analysis confirmed these results, with the global morphological pattern differing among species (*Pillai’s Trace* = 1.609, *F* = 16.822, d.f. = 22, *P* < 0.001), but not between the sexes (*Pillai’s Trace* = 0.321, *F* = 1.894, d.f. = 22, *P* = 0.067).

Further investigation of the wing set showed that *Siagona dejeani* and *Siagona jenissoni* are brachypterous (respectively 1.93±0.03 mm and 0.94±0.03 mm wing lengths), while *Siagona europaea* has long wings (8.01±0.05 mm) folded under the elytra, and can thus be considered a macropterous species presumably capable of flight.

## Discussion

The three *Siagona* species investigated presented sex and inter-specific differences. The sexes differ only in size: males of *Siagona dejeani* and *Siagona jenissoni* had significantly longer trochanters (relative to body length) than females, while in *Siagona europaea* females had wider heads than males.

These *Siagona* species are olfactory hunters and belong to the third group of nocturnal species described by Bauer and Kredler (1993), based on compound eye characteristics; laboratory recordings of activity of *Siagona europaea* have confirmed their nocturnal habit (Bauer et al. 2005). Nevertheless, eye parameters differed significantly among the species: the number of ommatidia is much higher in *Siagona europaea* (more than 500, see Table 2) than in the other two species (no more than 400 for *Siagona dejeani* and 300 for *Siagona jenissoni*). Presumably *Siagona europaea* has better visual capabilities than the others two species, even though all three species are nocturnal.

**Table 2.** Inter-specific differences in body and eye morphological characteristics (means and Standard Error of Means) in three species of *Siagona*. Kruskal-Wallis test results estimated with a permutation procedure (*PMonte Carlo*) are reported.<br/>

	Species						Kruskal-Wallis test		
	*Siagona dejeani*		*Siagona europaea*		*Siagona jenissoni*				
	Mean	SEM	Mean	SEM	Mean	SEM	Chi-Square	df	P*Monte Carlo*
Body length (mm)	23.08	0.17	11.44	0.16	14.25	0.09	52.569	2	<0.001
Antenna length (mm)	12.70	0.14	7.00	0.10	8.93	0.06	52.468	2	<0.001
Head width (mm)	5.07	0.05	2.58	0.03	3.25	0.03	52.469	2	<0.001
Number of ommatidia	405.95	14.23	546.75	19.10	315.60	6.07	44.612	2	<0.001
Eye surface (mm2)	0.06	0.01	0.01	0.00	0.04	<0.01	28.722	2	<0.001
Trochanter length (mm)	1.68	0.03	0.85	0.01	1.01	0.01	51.865	2	<0.001
Head height (mm)	2.78	0.05	1.31	0.03	1.78	0.04	50.937	2	<0.001
Thorax height (mm)	3.35	0.04	1.71	0.03	2.14	0.05	49.799	2	<0.001
Abdomen height (mm)	3.19	0.09	1.80	0.04	2.26	0.09	43.233	2	<0.001
Weighted antenna lenght	2.51	0.03	2.72	0.04	2.75	0.02	24.521	2	<0.001
Weighted trochanter length	0.07	0.00	0.07	0.00	0.07	0.00	9.586	2	0.007
Weighted ommatidia number	80.19	2.94	212.13	7.20	97.20	2.22	45.057	2	<0.001
Weighted head height	0.12	0.00	0.11	0.00	0.12	0.00	5.979	2	0.049
Weighted thorax height	0.15	0.00	0.15	0.00	0.15	0.00	2.977	2	0.228
Weighted abdomen height	0.14	0.00	0.16	0.00	0.16	0.01	9.667	2	0.007
Ommatidia density	20192.84	6895.16	68771.49	4740.88	9036.79	644.29	30.951	2	<0.001

*Siagona europaea* has a higher number of ommatidia, full-sized wings and a greater antenna vs. head ratio. These features are indicative of high dispersal powers, which is a good strategy for adapting to habitats such as pastures and fields, where natural or anthropogenic disturbances are frequent. Furthermore, it is likely that high powers of dispersal enhance the probability of finding new ant nests, which are often scattered rather than homogeneously distributed throughout the territory. Conversely, *Siagona dejeani* has shorter antennae, a lower number of ommatidia and smaller wings than the other two species. This is presumably related to a lesser need to search for a partner, as the beetles live in aggregation in which chemical cues easily allow males and females to meet.

In conclusion, the general morphometry of these three Mediterranean species of the genus *Siagona* is typical of beetles living in narrow spaces, presumably in darkness, for most of their life. As a consequence, eye morphology is well adapted to their habitat demands and to olfactory/tactile predation. Indeed, success in detecting ants or ant traces is assured by the complex sensory structure of the labial palps, which has been described in detail for *Siagona europaea* (Giglio et al. 2009). The antennae of male and female beetles are likely similar in their general structure in each of the three species (Giglio et al. 2007) and vary only in size. Conversely, some significant differences occur in the size of the wings, which are well developed only in *Siagona europaea*, the only species capable of flight. In this species, ecological demands are probably both for better vision for orientation in space and an enhanced antennal sensorial role in searching for a partner after dispersal by flight.

### Concluding remarks

**Figure 3.**
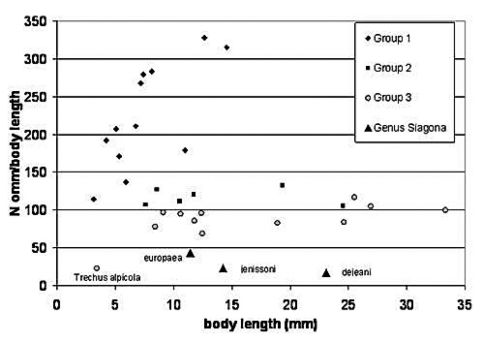
Ommatidial index versus body length in the three Bauer-Kredler groups of carabids and comparison with European species of *Siagona* (this study). Y-axis: mean number of ommatidia per body length in mm. In order of decreasing ommatidial index, **Group 1:** *Cicindela campestris*, *Cicindela hybrida*, *Elaphrus cupreus*, *Elaphrus riparius*, *Elaphrus ullrichi*, *Elaphrus aureus*, *Notiophilus biguttatus*, *Asaphidion flavipes*, *Asaphidion pallipes*, *Asaphidion caraboides*, *Bembidion quadrimaculatum*. **Group 2:** *Carabus granulatus*, *Agonum sexpunctatum*, *Poecilus cupreus*, *Poecilus versicolor*, *Carabus auratus*. **Group 3:** *Carabus problematicus*, *Carabus lefebvrei*, *Carabus coriaceus*, *Leistus rufomarginatus*, *Nebria brevicollis*, *Pterostichus nigrita*, *Carabus preslii*, *Abax parallelepipedus*, *Patrobus atrorufus*, *Pterostichus burmeisteri*, *Trechus alpicola*.

The carabid genus *Siagona* is a stenotopic ground dweller that preys on ants in the deep fissures of clayey soil. In Fig. 3 we compared the eye sizes of the three European species with the “ommatidial indices” of the three groups of Bauer and Kredler (1993). The first group includes diurnally active species, many of them true “visual hunters”, while the second group includes taxa with a less fixed or twilight activity period and the third includes nocturnally active carabids. The three *Siagona* species occupy a very unique position in this graph, similar to the more or less microphthalmic *Trechus alpicola* Sturm, 1825, a species living under stones in the Central Austrian Alps. The ommatidia/body length ratios of *Siagona jenissoni* and *Siagona dejeani* are particularly low (23 and 17, respectively), while the mean value of *Siagona europaea* is somewhat higher (43), indicating a pronounced adaptation to dark conditions of the habitat.

In conclusion, the European *Siagona* species exhibit a lifestyle thus far unknown in carabid beetles, i.e., a stenotopic adaptation to clayey soils of tropical and subtropical climates marked by a long dry season. The adults probably enter fissures in the clay at the beginning of the dry phase and are able to exploit the rich trophic resources (ant workers and perhaps ant brood) in this three-dimensional subterranean space.
